# Importance of clinical profiling to determine excess mortality in prolactinomas: insights from a large, registry-based cohort

**DOI:** 10.1007/s11102-026-01739-w

**Published:** 2026-09-03

**Authors:** Tarik Ziyad Tarik Shwaish, Davide Gori, Claudio Sartori, Federica Guaraldi

**Affiliations:** 1 Department of Medical Sciences, Division of Endocrinology, Diabetes and Metabolism, University of Turin, Turin, 10126 Italy; 2 Department of Biomedical and Neuromotor Sciences, University of Bologna, Bologna, 40126 Italy; 3 Department of Computer Science and Engineering, University of Bologna, Bologna, 40126 Italy; 4 Pituitary Unit, IRCCS Istituto delle Scienze Neurologiche di Bologna, Bologna, 40139 Italy; 5 Department of Biomedical and Neuromotor Sciences, University of Bologna, Via San Giacomo, 12, Bologna, 40126 Italy

**Keywords:** Prolactinoma, Pituitary adenoma, Mortality, SEER

## Abstract

**Purpose:**

Population-based studies overall report no excess mortality among patients with prolactinoma compared with the general population. However, since prolactinomas are clinically heterogeneous, mortality may not be uniform across data-driven patient profiles.

**Methods:**

We analyzed 3,378 adult prolactinoma cases (26,118 person-years) from the Surveillance, Epidemiology, and End Results database (2004–2022). Standardized mortality ratios (SMRs) were calculated using US life tables matched by age, sex, calendar year, race/ethnicity, and geography. Among 2,481 patients with available tumor size (18,097 person-years), unsupervised clustering (Fuzzy C-Means) based on age, tumor size, and sex defined patient profiles. Excess mortality ratios (EMRs) compared observed-to-expected mortality across profiles, adjusting for race, calendar year, surgery, and radiotherapy.

**Results:**

Prolactinoma was associated with significant excess all-cause mortality (SMR 1.21, 95% CI 1.03–1.41). Two profiles emerged: SAYF (Small and Young, Female-predominant; 63%) and LOOM (Large or Old, Male-predominant; 37%). Among patients with tumor size data, mortality was increased in LOOM (SMR 1.36, 95% CI 1.11–1.65) and reduced in SAYF (SMR 0.48, 95% CI 0.22–0.90), with significant between-profile heterogeneity (EMR 2.92, 95% CI 1.44–5.90; *p* = 0.003). Results were consistent in sensitivity analyses with imputed data, although the survival advantage in SAYF was attenuated and no longer statistically significant.

**Conclusion:**

Prolactinoma captured in a population-based tumor registry was associated with excess all-cause mortality, with heterogeneity across patient profiles. An older, male-predominant profile with larger tumors showed excess mortality, whereas a younger, female-predominant profile with small tumors showed directionally lower mortality. These hypothesis-generating findings warrant further investigation into the underlying biological and contextual factors.

**Supplementary Information:**

The online version contains supplementary material available at https://doi.org/10.1007/s11102-026-01739-w.

## Introduction

Prolactinomas account for nearly half of clinically recognized pituitary adenomas, with an estimated prevalence 47–62 cases/100,000 persons, and incidence of 3–5 new cases/100,000/year. Women are affected 3–10 times more than men and typically present with microprolactinomas (size < 10 mm) between the age of 25 to 34 years old, while men are often diagnosed after the age of 50 with larger and more aggressive tumors [1]. Most population-based studies have reported no excess mortality in prolactinomas compared with the general population [2–4]. However, these tumors are heterogeneous, and it is unclear whether this view applies uniformly to all patients or varies across distinct patient profiles.

In other pituitary adenoma subtypes, increased mortality is well established. Acromegaly and Cushing disease confer an elevated death risk, and non-functioning adenomas have also been associated with excess mortality, even in the absence of documented hypopituitarism [5–7]. A nationwide Korean study found that, unlike other pituitary adenoma subtypes, prolactinoma was not related to excess mortality [3]. A recent nationwide Israeli study similarly reported no overall excess mortality in patients treated with dopamine agonists [2]. On the other hand, the Scottish PROLEARS cohort study reported excess deaths in macroadenomas but not microadenomas, though it was limited by small sample size [8]. Notably, neither the Korean nor Israeli study included tumor size, and none of these studies assessed interactions between age and sex, particularly around menopause, a central determinant of female prolactinoma phenotype.

Clinically, prolactinoma presentation varies by sex and age [1]. Premenopausal women commonly present with amenorrhea and galactorrhea and may experience spontaneous remission after menopause or pregnancy. Men more frequently present with mass effects, hypopituitarism, and dopamine agonist resistance. Postmenopausal women resemble men in tumor characteristics and outcomes, suggesting a biologically relevant transition around menopause [9].

We hypothesized that excess mortality in prolactinoma is not uniform but varies across patient profiles defined by sex, age, and tumor size. To test this, we applied unsupervised learning to a large cohort from the Surveillance, Epidemiology, and End Results (SEER) database, representing approximately 26.5% of the US population, and compared excess mortality relative to the general population across the identified subgroups. This study did not aim at identifying causal factors or predictors of increased risk, but rather at providing a large-scale analysis of survival as an observed feature of patient profiles.

## Methods

### Data source and inclusion criteria

Adult-onset prolactinoma cases, diagnosed between January 1, 2004, and December 31, 2022, were retrieved from the Surveillance, Epidemiology, and End Results (SEER) Research Database (17-registry dataset, November 2024 submission) [10]. The registries included were San Francisco-Oakland, Connecticut, Hawaii, Iowa, New Mexico, Seattle-Puget Sound, Utah, Metropolitan Atlanta, San Jose-Monterey, Los Angeles, Alaska Natives, Rural Georgia, Greater California (excluding San Francisco-Oakland, San Jose-Monterey, and Los Angeles), Kentucky, Louisiana, New Jersey, and Greater Georgia.

Inclusion criteria were primary site code C75.1 (pituitary gland) and ICD-O-3 histological code 8271/0 (benign prolactinoma) [11]. Exclusion criteria were death-certificate-only or autopsy-only reports, patients recorded as alive with no survival time, pediatric cases (age at diagnosis < 18 years), and patients aged ≥ 90 years at diagnosis; the latter were excluded because SEER does not provide exact age for this population, making relative mortality and clustering analyses unreliable.

### Variable definition and encoding

Age (years) was recorded at the time of diagnosis. Tumor size (millimeters, mm) was the maximum diameter documented at initial assessment. Surgical treatment was classified as ‘no surgery’ (site-specific code 00), ‘gross total resection’ (GTR; codes 40, 41, and 60), and ‘subtotal resection’ (STR; codes 10–14, 20–27, 30, and 50), while radiation treatment was considered as a binary variable (received vs. not received). In SEER, patients who are not known to have received radiation are grouped with those known to have not received the treatment. Mutually exclusive race/ethnicity groups were non-Hispanic (NH) White, NH Black, NH American Indian/Alaskan Native, NH Asian and Pacific Islander, NH Unknown, and Hispanic.

### Descriptive analysis

Categorical variables were reported as number of cases (n) and percentages (%). Continuous variables were reported as median and interquartile range (IQR) because they followed a non-normal distribution (according to Q-Q plots). Group comparisons were performed via the Fisher’s exact and Mann-Whitney U tests for categorical and continuous variables, respectively. The Freeman-Halton extension of Fisher’s exact was used for tables larger than 2 × 2. Significance was defined as two-tailed *p* < 0.05.

### Preliminary mortality analysis

The standardized mortality ratio (SMR) with 95% CI: was computed as the ratio between observed and expected deaths calculated using the US SES/Geography/Race Annual Life Tables matched by sex, age, race/ethnicity, calendar year, and geography [12, 13]. SMR was calculated for the entire cohort and after stratification by sex, tumor size quartiles, age quartiles, surgical resection, radiation therapy, and race/ethnicity, and for females, menopause (defined as age ≥ 52 years). The Cochran Q test was used to compare SMR across these subgroups.

### Clustering-based mortality analysis

Fuzzy C-Means (FCM) clustering was performed based on age, tumor diameter, and sex [14]. Records missing any of these variables were excluded from the clustering analysis. Age and tumor size were scaled by Z-score. Sex was encoded as a binary (0/1) variable, such that a difference in sex was equivalent to a 1-standard-deviation difference in age or tumor size. The number of clusters (2–5) was selected by the highest mean silhouette score in 5-fold cross-validation, and the fuzzifier (*m* = 3.7) was derived by the Schwämmle and Jensen (2010) method from the number of input variables (*D* = 3) and the clustering sample size [15]. Cluster boundaries in age-tumor-size space were visualized as the contour of equal membership, and distributions were compared across clusters with descriptive statistics.

Within-cluster SMRs assessed survival relative to the general population. To formally test the association between cluster assignment and excess mortality, a Poisson regression with robust standard errors was fitted [16], and excess mortality ratios (EMRs) with 95% CIs were calculated both crude and adjusted for race/ethnicity, calendar year, surgery (received vs. not received, with unknown surgical status treated as not received), and radiation therapy.

Cluster stability was assessed using a resampling framework based on 50 iterations of 50% test/training set splits [17]; within each iteration, test-set assignments from test-fitted models were compared with assignments from training-set centroids, and concordance was quantified by the Adjusted Rand Index (ARI) with 95% CI.

Clustering was chosen because age, sex, and tumor size are strongly interrelated and jointly define clinically meaningful profiles, whereas traditional regression estimates marginal effects conditional on other variables, which is unstable under collinearity or low event counts, and may miss patterns in the joint distribution of predictors. Clustering thus summarized the multidimensional predictor space into coherent subgroups for evaluating observed versus expected mortality. The procedure was not intended to identify independent predictors of mortality, which would be conceptually circular, as age and sex contribute both to profile definition and, via life table matching, to expected mortality, but rather to define empirically derived profiles for assessing whether excess mortality differed across them.

FCM was preferred over hard clustering because biological phenotypes rarely form discrete categories: rather than assigning each patient to a single cluster, FCM yields continuous membership values across clusters, better reflecting an underlying phenotypic spectrum [18]. Hard labels (from convergence membership scores) were used for the main analysis for interpretability, with a sensitivity analysis assessing the association between continuous scores and excess events.

Age and tumor size were not transformed prior to scaling; consequently, extreme values contributed more strongly to pairwise dissimilarity, and clustering reflected the marginal distributions of the inputs, as prespecified to preserve observed data structure. Robustness to extreme tumor sizes was assessed using Winsorization at the 99th percentile, and the contribution of the categorical variable (sex) was evaluated by comparing the full three-variable model with a reduced two-variable model including age and tumor size only, using both *m* = 3.7 (as specified in the main analysis) and *m* = 6.8 (recalculated for the two-variable input space).

Because prolactinomas can, exceedingly rarely, directly cause death, a sensitivity analysis recalculated SMRs and EMRs after excluding deaths classified under the “Brain, CNS Other, and Intracranial Gland Neoplasm (Benign and Borderline)” category of the SEER “Cause of Death to Site Recode ICD-O-3 2023 Revision” variable. This criterion was appropriate because a preliminary analysis showed that no individual with this cause of death had a recorded additional benign or borderline CNS or intracranial neoplasm beyond prolactinoma.

All cases excluded from clustering had missing tumor size, with none excluded for missing age or sex. To assess complete-case bias, patients with and without recorded tumor size were compared descriptively, with a standardized mean difference (SMD) < 0.10 considered small [19]. Robustness to tumor size missingness was further evaluated by classification and regression tree (CART)-based multiple imputation (pre-specified maximum depth 5): thirty datasets were imputed using age, sex, race/ethnicity, surgery, radiotherapy, survival time, vital status, and life-table-derived expected survival; patients were assigned to the complete-case clusters using the original centroids, and cluster-specific SMRs and EMRs were estimated per dataset and pooled by Rubin’s rules.

### Software and reporting guidelines

SEER*Stat 8.4.5 (National Cancer Institute, Bethesda, MD, USA; released April 2024) was used to retrieve data and calculate per-patient expected survival [20]. Analysis and visualization were performed in KNIME 5.4.1 (KNIME AG, Zurich, Switzerland; released February 2025) with Python 3.13.1 integration [21]. This study is reported in accordance with the Strengthening the Reporting of Observational Studies in Epidemiology (STROBE) guidelines for cohort studies [22].

## Results

### Study population

**Fig. 1:**
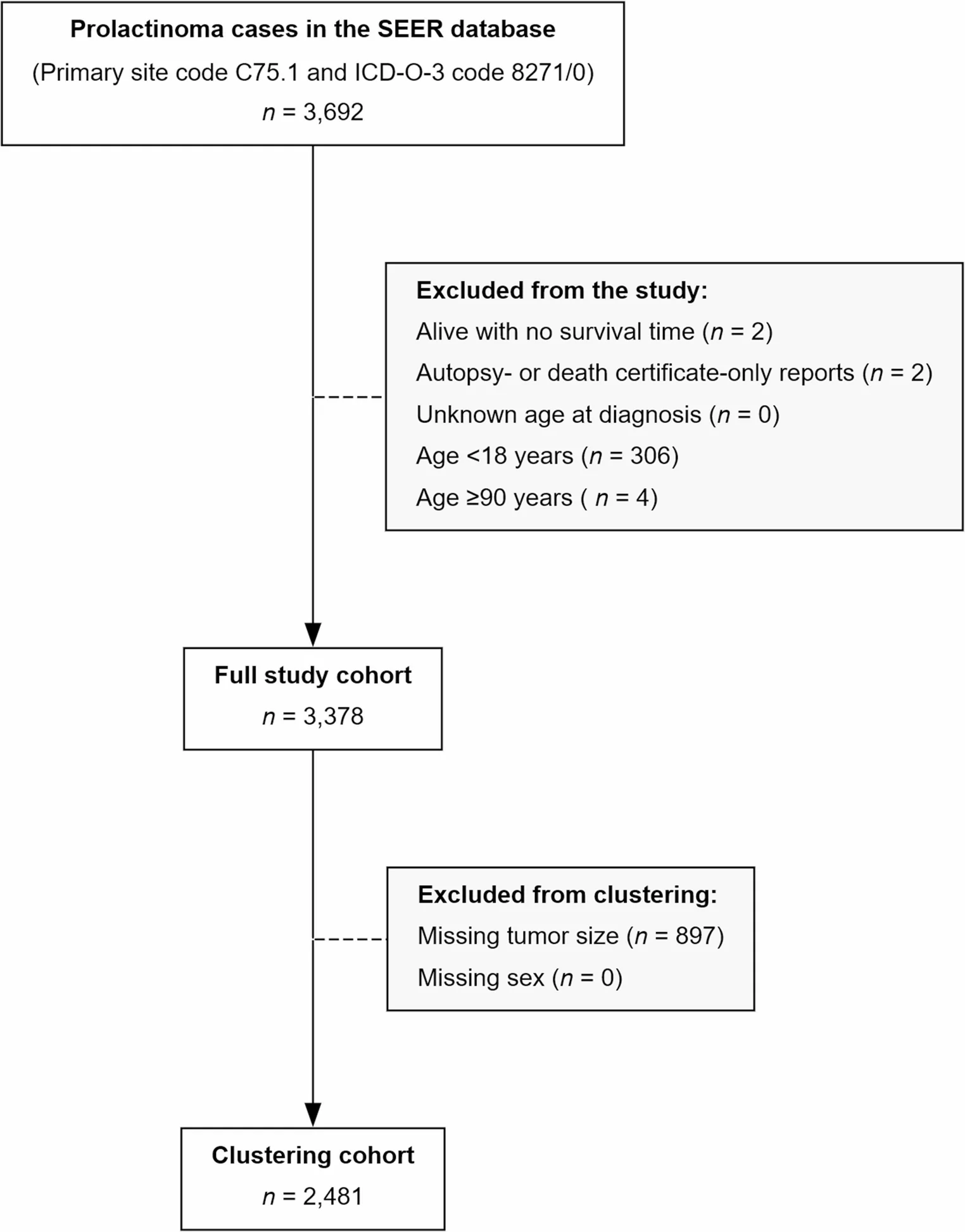
Flow diagram of cohort selection from the surveillance, epidemiology, and end results (SEER) database. *n*, number of patients; ICD-O-3, international classification of diseases for oncology, third edition

The study cohort derivation procedure is summarized in Fig. 1. The full cohort comprised 3,378 patients (26,118 person-years). After exclusion of 897 patients with missing tumor size, 2,481 individuals (18,097 person-years) remained for the clustering analysis.

Comparison of patients with and without available tumor size data (Supplementary Table S1) showed that tumor size was more often documented in more recent calendar years (SMD = 0.342; *p* < 0.001) and among surgically treated patients (SMD = 0.156; *p* < 0.001). Differences in age (*p* = 0.027), sex (*p* = 0.048), race/ethnicity (*p* = 0.104), and radiotherapy (*p* = 0.817) were small in magnitude (SMD < 0.10). Excess mortality did not differ according to tumor size missingness (SMR 1.185 [95% CI: 0.976–1.426] for size known vs. 1.263 [95% CI: 0.954–1.640] for size missing; *p*-heterogeneity = 0.697). Among deaths with a known cause of death (*n* = 162/168 deaths in the full cohort), the proportion attributed to prolactinoma itself showed no significant difference between patients with available and missing tumor size (7.4% [8/108] vs. 5.6% [3/54], respectively; *p* = 0.753). Causes of death of the complete cohort, stratified by tumor missingness status, are presented in Supplementary Table S2.

### Baseline cohort characteristics

Table 1 presents the baseline characteristics of the full study population (*n* = 3,378). Females constituted 62.8% (*n* = 2,122) of the study cohort and males 37.2% (*n* = 1,256). The median age at diagnosis was 36 years, with male tumors diagnosed at a significantly later age compared with females (median 45 vs. 32 years, *p* < 0.001). The median tumor size was 10 mm, with significantly larger tumors diagnosed in males compared with females (median 21 vs. 7 mm, *p* < 0.001).

**Table 1:** Cohort characteristics stratified by sex. Continuous variables are described as median (interquartile range) and compared using Mann-Whitney U test. Categorical variables are described as percentage (frequency) and compared using fisher’s exact test

Variable	Level	Overall (100% [*n* = 3,378])	Female (62.8% [*n* = 2,122])	Male (37.2% [*n* = 1,256])	*p*-value
Age (years)		36.0 (28.0–48.0)	32.0 (26.0–40.0)	45.0 (34.8–57.0)	< 0.001
Tumor size (mm)		10.0 (6.0–22.0)	7.0 (5.0–12.8)	21.0 (10.0–33.0)	< 0.001
Surgery	No	85.8% (2880/3,358)	88.3% (1862/2,108)	81.4% (1,018/1,250)	< 0.001
	Yes	14.2% (478/3,358)	11.7% (246/2,108)	18.6% (232/1,250)	
Radiation (received)	No	99.3% (3354/3,378)	99.6% (2113/2,122)	98.8% (1,241/1,256)	0.018
	Yes	0.7% (24/3,378)	0.4% (9/2,122)	1.2% (15/1,256)	
Race/ethnicity	White	51.6% (1701/3,299)	46.8% (972/2,077)	59.7% (729/1,222)	< 0.001
	Hispanic	23.5% (774/3,299)	24.7% (512/2,077)	21.4% (262/1,222)	
	Black	14.5% (480/3,299)	16.7% (347/2,077)	10.9% (133/1,222)	
	Asian or Pacific Islander	8.9% (295/3,299)	10.0% (208/2,077)	7.1% (87/1,222)	
	American Indian/Alaska Native	1.5% (49/3,299)	1.8% (38/2,077)	0.9% (11/1,222)	

^a^ Excluding 20 patients (0.6%) with unknown surgical history

^b^ Excluding 79 patients (2.3%) with unknown (non-Hispanic) race

In terms of treatment, 14.2% of the study population underwent surgical resection and 0.7% radiation therapy. Both treatments were performed more frequently in males (surgery *p* < 0.001, radiation *p* = 0.018). Among patients who underwent surgery, there were no sex-related differences in resection margins, with STR reported in 88.8% (215/242) of females and 88.2% (202/229) of males (*p =* 0.517).

The most represented ethnicities were White (51.6%), Hispanic (23.5%), and Black (14.5%). There were significant sex differences in racial distribution (*p* < 0.001), with a lower proportion of White individuals and a higher proportion of every other ethnicity among females compared with males.

### Mortality analysis and assessment of heterogeneity across prespecified strata

Overall, mortality in patients with prolactinoma was significantly higher compared with the general population (SMR 1.210, 95% CI: 1.034–1.408, *p* = 0.018). Among 3,378 patients followed for a median of 85.0 months (IQR 41.0–139.0), there were 168 deaths that occurred at a median of 58.0 months (IQR 26.8–112.8) after diagnosis, compared with 138.833 expected deaths in a matched general population. **Supplementary Table S3** shows detailed stratified SMR calculations.

There was a progressive increase in SMR across tumor size quartiles (*p*-heterogeneity = 0.015): Q1 (1–6 mm) 0.554, Q2 (6–10 mm) 0.901, Q3 (10–22 mm) 1.187, Q4 (22–115 mm) 1.573, with only the largest tumors (Q4) demonstrating statistically significant excess mortality (*p* = 0.002).

There was an apparent dose-response relationship between surgical resection and excess mortality, but heterogeneity did not reach significance (*p*-heterogeneity = 0.067); SMR increased from 1.130 in non-operated patients to 1.398 for STR and 2.628 for GTR, with only patients who underwent a GTR showing a statistically significant excess mortality (*p* = 0.039). Radiotherapy was associated with significant heterogeneity (*p*-heterogeneity = 0.046), with higher excess mortality among patients who received the treatment (SMR 3.273 vs. 1.192), although neither stratum reached significance.

SMR also showed a significant difference per race/ethnicity (*p*-heterogeneity = 0.002). Excess mortality reached significance among Hispanic (SMR 1.746, *p* = 0.005) and Asian or Pacific Islanders (SMR 2.848, *p* < 0.001), but not among White, Black, or American Indian/Alaska Native patients.

Age quartiles showed non-significant heterogeneity (*p*-heterogeneity = 0.094); there was no clear dose-response relationship, with only the oldest age quartile (48–89 years) demonstrating a statistically significant excess mortality (SMR 1.345, *p* = 0.001). Excess mortality also showed no statistically significant heterogeneity in relation to sex (*p*-heterogeneity = 0.208; SMR 1.369 and 1.123 among females and males, respectively) or the menopausal status among females (*p*-heterogeneity = 0.375; SMR 1.514 and 1.217 among post-menopausal and pre-menopausal women, respectively).

### Clustering-based analysis

FCM clustering based on patient proximity in sex, age, and tumor size (among the clustering cohort [*n* = 2,481]) identified two patient subgroups, which were designated SAYF (Small and Young, Female-predominant; *n* = 1,572 [63.4%]) and LOOM (Large or Old, Male-predominant; *n* = 909 [36.6%]). The solution demonstrated strong reproducibility across data subsets, with ARI of 0.94 (95% CI: 0.94–0.95).

**Fig. 2:**
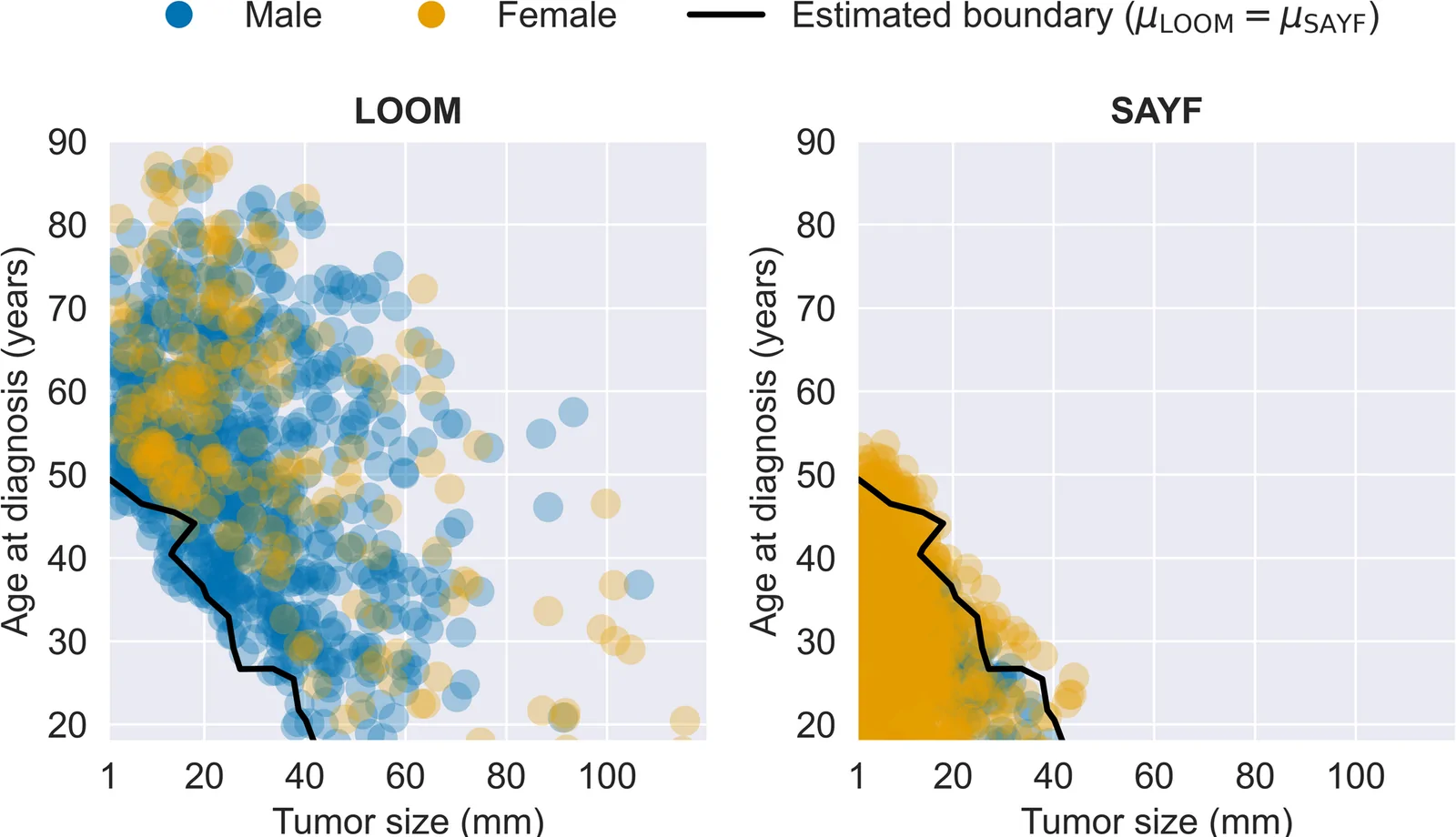
Scatter plot of patients in cluster LOOM (Large or old, male-predominant; left) and SAYF (Small and young, female-predominant; right). Tumor size (mm) is shown on the x-axis and age at diagnosis (years) on the y-axis. Each circle represents one patient; males are shown in blue and females in orange. The cubic fitted boundary between clusters is overlaid on both plots

Figure 2 shows a scatter plot for patients in SAYF and LOOM clusters, colored by sex. The plot shows tumors that were larger or diagnosed in later age were assigned to cluster LOOM, which was predominated by males, while SAYF comprised small tumors in the young and was female predominant. The contour separating the two clusters in the two-dimensional space of tumor size and age intersected the age axis at 50 years (tumor size = 1 mm) and the tumor size axis at 41 mm (age = 18 years), indicating that the cluster transition occurred along a boundary linking the end of reproductive age with the clinical definition of giant tumors (diameter > 40 mm [1]). LOOM patients had a statistically significant higher rate of surgery (*p* < 0.001) and radiation (*p* = 0.001). Table 2 contains a detailed descriptive cluster analysis.

**Table 2:** Cohort characteristics stratified by cluster. Continuous variables are described as median (interquartile range) and compared using Mann-Whitney U test. Categorical variables are described as percentage (frequency) and compared using fisher’s exact test

Variable	Level	LOOM (36.6% [*n* = 909])	SAYF (63.4% [*n* = 1,572])	*p*-value
Sex	Female	24.0% (218/909)	83.7% (1,316/1,572)	< 0.001
	Male	76.0% (691/909)	16.3% (256/1,572)	
Age (years)		52.0 (41.0–63.0)	31.0 (25.0–37.0)	< 0.001
Tumor size (mm)		25.0 (15.0–39.0)	7.0 (5.0–11.0)	< 0.001
Surgery ^a^	No	79.1% (716/905)	87.4% (1,370/1,567)	< 0.001
	Yes	20.9% (189/905)	12.6% (197/1,567)	
Radiation	No	98.6% (896/909)	99.7% (1568/1,572)	0.001
	Yes	1.4% (13/909)	0.3% (4/1,572)	
Race ^b^	White	57.9% (518/894)	48.2% (739/1,533)	< 0.001
	Hispanic	21.8% (195/894)	24.5% (376/1,533)	
	Black	12.4% (111/894)	14.4% (221/1,533)	
	Asian or Pacific Islander	6.6% (59/894)	11.1% (170/1,533)	
	American Indian/Alaska Native	1.2% (11/894)	1.8% (27/1,533)	

^a^ Excluding 9 patients (0.4%) with unknown surgical history

^b^ Excluding 54 patients (2.2%) with unknown (non-Hispanic) race

Mortality analysis showed observed deaths in SAYF were significantly fewer than expected (SMR 0.476, 95% CI: 0.218–0.904, *p* = 0.019), whereas LOOM patients experienced a statistically significant excess mortality (SMR 1.362, 95% CI: 1.112–1.652, *p* = 0.003). The multivariable EMR, accounting for differences in race, diagnosis year, surgery, and radiation, was 2.917 (95% CI: 1.443–5.896, *p* = 0.003), confirming an association between cluster membership and excess death compared to expectations in a matched general population. These mortality findings are detailed in Table 3.

**Table 3:** Follow-up and mortality outcomes stratified by cluster

Measure	SAYF (*n* = 1,572)	LOOM (*n* = 909)
Median follow-up months (IQR) ^a^	79.0 (38.0–135.0)	78.0 (36.0–131.0)
Observed deaths (n)	9	103
Median months to death (IQR)	106.0 (21.0–133.0)	47.0 (22.0–104.5)
Expected deaths (n)	18.890	75.618
SMR (95% CI) ^b^	0.476 (0.218–0.904)	1.362 (1.112–1.652)
*p*-value	0.019	0.003
Crude EMR (95% CI)	Reference	2.859 (1.445–5.658)
*p*-value	-	0.003
Adjusted EMR (95% CI) ^c^	Reference	2.917 (1.443–5.896)
*p*-value	-	0.003

^a^ *p* = 0.355 (Mann Whitney U test)

^b^ *p* = 0.003 (Cochran Q test for heterogeneity)

^c^ Adjusted for race, calendar year, surgery, and radiation treatment

*IQR* interquartile range, *SMR* standardized mortality ratio, *EMR* excess mortality ratio, 95% CI 95% confidence interval

Although the relative survival differences were significant, the absolute differences were modest: compared to a matched general population, LOOM patients experienced 4.21 additional deaths per 1,000 person-years (95% CI: 1.30–7.59), while SAYF patients 0.85 fewer deaths per 1,000 person-years (95% CI: 0.16–1.27). Multivariable regression showed that the membership to LOOM was associated with 3.12 additional deaths per 1,000 person-years (95% CI: 0.72–7.97), after accounting for race, diagnosis year, surgery, and radiation.

**Fig. 3:**
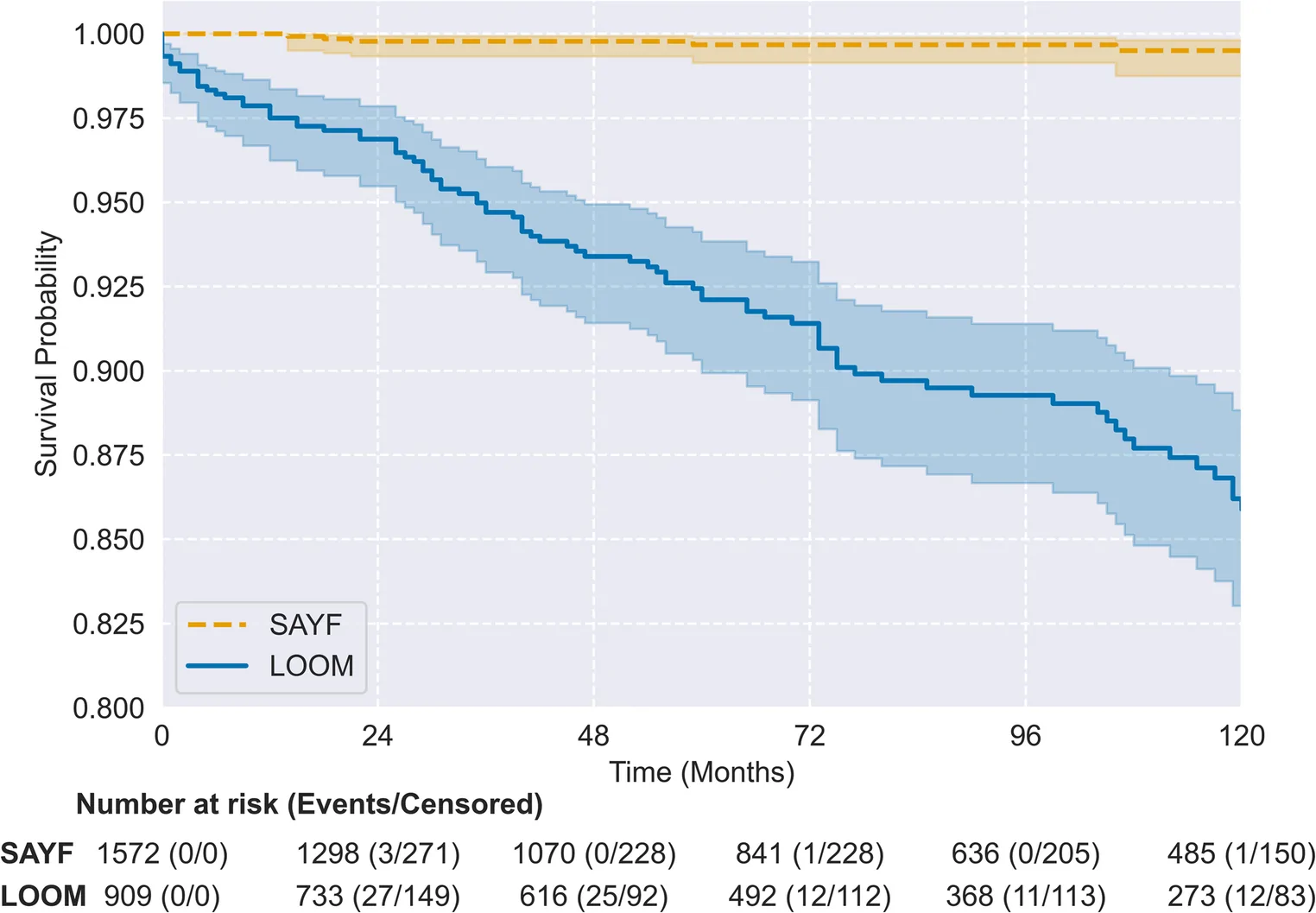
Kaplan-Meier survival curves stratified by cluster. Numbers at risk are shown at 24-month intervals below the plot, along with patients who died or were last censored in the preceding interval

Figure 3 shows a Kaplan-Meier curve of the two clusters. Plotted survival differences are purely descriptive and represent raw deaths, not compared to the general population

### Clustering sensitvity analyses

In a secondary Poisson model treating cluster membership as a continuous variable, higher LOOM membership was confirmed to be associated with excess death (univariable EMR *p* = 0.002; multivariable EMR *p* = 0.006; Supplementary Fig. S1).

Sensitivity analyses based on hard labels confirmed robust cluster structures and mortality patterns (Supplementary Table S4). Within the clustering cohort, exclusion of 8 cases where cause of death pointed to prolactinoma (all assigned to LOOM; 5 males and 3 females; age range 29–81 years; tumor size range 23–68 mm; median time from diagnosis to death 3.5 months [IQR 0.5–26]), Winsorization of tumor size at the 99th percentile, and clustering based on age and tumor size alone (with both fuzzifier settings *m* = 3.7 and *m* = 6.8) all yielded highly stable cluster assignments (ARI ≥ 0.83), confirming mortality patterns observed in the main analysis. Notably, the model excluding sex as input variable showed persistent sex-related differences (*p* < 0.001 for both *m*).

When original centroids were applied to the imputed full cohort (*n* = 3,781), results showed persistent mortality heterogeneity (adjusted EMR 1.763, 95% CI 1.091–2.849, *p* = 0.021); LOOM showed a statistically significant excess mortality similar to the main analysis (SMR 1.316, 95% CI 1.120–1.547, *p* < 0.001), whereas SAYF showed a directionally consistent but non-significant survival advantage (SMR 0.769, 95% CI 0.497–1.188, *p* = 0.236).

## Discussion

This is, to our knowledge, the first large-scale population-based study to report statistically significant excess all-cause mortality in prolactinoma. Among 3,378 adults followed for 26,118 person-years, observed deaths exceeded those expected from a matched general population (SMR 1.21, 95% CI 1.03–1.41). Excess mortality was heterogeneous by patient profile: a young female profile with smaller tumors showed a survival advantage, whereas an older, male-predominant profile with larger tumors was associated with excess mortality.

The aggregate SMR contrasts with the two largest prior investigations of prolactinoma mortality, from Korea and Israel [2, 3]. The Korean SMR was directionally consistent with our findings, with prolactinoma showing the lowest SMR among pituitary adenoma subtypes (SMR 1.10, 95% CI 0.93–1.30) [3]. The Israeli study, in dopamine agonist-treated hyperprolactinemia, found no difference from matched controls (HR 0.972, 95% CI 0.829–1.139) [2]. However, in contrast to both our study and the Korean study, the Israeli study matched patients to controls not only by demographics but also by BMI, as it aimed to isolate disease effect rather than describe mortality among the diagnosed population. As patients with prolactinoma have an increased risk of obesity [23], this may have led to a more conservative estimate of excess mortality.

Several structural features distinguish this study from prior work and may account for these discrepancies. First, although SEER is population-based and includes benign brain and central nervous system tumors from 2004 inwards, selection bias cannot be ruled out, both from ascertainment of more severe cases (rather than community-treated hyperprolactinemia) and from prior tumor history. Indeed, the Korean study was larger (*n* = 8,871) yet recorded fewer deaths (*n* = 136) than the current study (*n* = 168). Regarding possible enrichment for surgically treated cases, 14.2% of our patients underwent surgery, but direct comparison is impossible, as neither prior study reported this proportion. Second, unlike the present study, neither the Korean nor the Israeli study was restricted to adults; the resulting differences in age composition may contribute to the divergent estimates, given the strong age dependence of both prolactinoma phenotype and background mortality. Third, our US-based cohort inherently differs from those of Korea and Israel in setting (e.g., healthcare system) and composition (e.g., racial/ethnic groups, comorbidities). While SMR varied significantly across racial/ethnic groups, this must be interpreted cautiously given residual confounding, small subgroup death counts, and known limitations of life tables in some racial/ethnic populations [12, 13].

Comparisons of SMR across settings, disease entities, and registries warrant caution. Although the aggregate SMR of 1.21 lies within a similar numerical range as acromegaly in Sweden (SMR 1.3, 95% CI 1.1–1.5) and Italy (SMR 1.7, 95% CI 1.4-2.0), and non-functioning pituitary adenoma in a recent meta-analysis (SMR 1.6, 95% CI 1.2-2.0) [6, 7, 24], this may not be interpreted as evidence of equivalent biological severity; it reflects prolactinoma mortality in the cohort captured by this national tumor registry. Notably, SEER ICD-O-3 coding classifies all pituitary adenomas under a unified histology code (except prolactinoma), limiting direct internal comparisons across adenoma subtypes.

Among patients with available tumor size, unsupervised clustering on age, tumor size, and sex identified two stable, clinically coherent profiles with distinct mortality. The SAYF cluster (63.4% of patients) experienced fewer deaths than expected (SMR 0.48, 95% CI 0.22–0.90), whereas the LOOM cluster (36.6%) showed significant excess mortality (SMR 1.36, 95% CI 1.11–1.65). Because expected mortality was matched for age and sex (and geography, race/ethnicity, and calendar year), the cluster differences reflect deviations beyond the matched demographic structure; nevertheless, because age and sex contribute both to cluster definition and, via life table matching, to expected mortality, these SMRs are best read as descriptive characteristics of population-level profiles rather than causal effects. The multivariable regression adjusting for race, calendar year, surgery, and radiation (EMR 2.92, 95% CI 1.44–5.90) likewise does not represent an age- and sex-independent effect, since cluster identity is itself partly defined by age and sex. This divergence offers a possible explanation for the aggregate neutrality reported previously [25]: the survival advantage of SAYF, the majority profile, may have counterbalanced or diluted the excess mortality in LOOM, yielding a neutral overall SMR.

The robustness of survival divergence differed between the two clusters. In the sensitivity analysis applying cluster centroids to multiply-imputed full-cohort datasets, the between-cluster mortality heterogeneity (assessed by EMR) was confirmed. While excess mortality in LOOM remained statistically significant, the survival advantage in SAYF was attenuated and no longer significant, although directionally consistent with the main analysis. This asymmetry may at least partly reflect the low number of deaths in SAYF combined with uncertainty in imputed tumor size. Accordingly, the survival advantage in SAYF is best interpreted as directional rather than definitive.

The boundary separating the clusters aligned with established clinical thresholds: approximately 50 years, corresponding to the end of the reproductive period, and approximately 40 mm, the definition of giant adenomas. Tumors in older individuals were assigned to LOOM, along with large, male-predominant tumors at younger ages, whereas SAYF mainly contained small tumors in reproductive-aged females. This is consistent with the well-described attenuation of sex-related differences in prolactinoma phenotype after menopause, attributed to estrogen-dependent mechanisms in premenopausal women [26–29]. Because variables were not transformed prior to clustering, these boundaries may partially reflect the underlying distributions, whereby values in distributional tails are more strongly separated from the more common phenotype.

Excess mortality in LOOM likely reflects both direct and indirect mechanisms. All deaths directly attributed to prolactinoma occurred in this cluster, meaning that direct prolactinoma lethality, which remains exceedingly rare, was limited to this profile [30, 31]. Excess mortality persisted after excluding these cases, implicating indirect pathways. Two such pathways are plausible. First, larger tumors are associated with a higher burden of chronic hypopituitarism related either to the tumor itself (mass effect), or iatrogenic, related to needing multiple treatments each potentially leading to pituitary damage (high-dose medical therapy potentially leading to pituitary apoplexy, one or more extended surgeries, and radiation therapy) [1, 32–36]. Male prolactinomas, which were dominant in LOOM, are characterized by long-standing and at times irreversible hypogonadism, which may increase susceptibility to metabolic and cardiovascular alterations [32, 37, 38]. Indeed, the Israeli study identified age, BMI, hypertension, diabetes mellitus, ischaemic heart disease, and congestive heart failure as independent mortality predictors in hyperprolactinemia [2]. Second, some LOOM tumors in older patients may be incidentalomas, with death related to the primary imaging indication, such as neurocognitive changes or cerebrovascular events [39]. However, prolactin-secreting incidentalomas are uncommon, representing only 8–16% of incidentaloma diagnoses [39].

The lower mortality observed in SAYF, though not confirmed in imputed datasets, still warrants careful consideration, as it may reflect healthy-user effects (a combination of ascertainment bias and confounding by healthcare utilization) and lead-time bias (due to earlier detection of the tumors), while not excluding true biological protection in this subpopulation. Premenopausal women with small tumors, who constituted most of SAYF, may be more likely to seek medical attention for menstrual disturbances and to be diagnosed when healthcare access is adequate, consistent with a reported inverse association between prolactinoma size and socioeconomic status in the United States [40]. Increased healthcare utilization after diagnosis in otherwise healthy individuals may further confound outcomes by facilitating earlier detection and management of competing health risks [1, 41].

Although improved survival in a tumor population may seem counterintuitive, it is a well-documented phenomenon in many early-stage tumors, including thyroid, prostate, breast, and melanoma [42]. Israeli data show that patients with dopamine agonist-treated hyperprolactinemia achieving prolactin normalization have better survival than matched controls, while those who do not normalize do not [2]. Whether this reflects a genuine biological effect or residual confounding remains uncertain [43]. While the Israeli study was able to explore relationships between disease activity, treatment response, comorbidities, and mortality, such analyses are not possible in SEER since the database lacks detailed clinical data.

Although two clusters represented the optimal mathematical solution, they should be seen as a phenotypic spectrum rather than a strict dichotomy. FCM models this continuum by outputting membership values, but we used hard labels for interpretability of patient distribution and survival [14, 18]. A model using continuous LOOM-versus-SAYF membership scores rather than hard labels confirmed the association of the LOOM profile with excess death.

These results offer a possible reconciliation between studies reporting increased cardiovascular burden and those reporting no excess mortality in prolactinoma [3, 44–47]. Cardiovascular disease was the most frequent cause of non-prolactinoma-related death in our cohort, and the Korean cohort reported significantly increased incidences of hemorrhagic stroke, ischemic stroke, and myocardial infarction [3]. Consistent with the dilution mechanism above, prolactinoma-associated cardiovascular risk may translate into excess mortality only in some patients while remaining masked at the aggregate level. Future studies assessing cardiovascular-specific mortality or event incidence across patient profiles would be informative.

Our results extend those of the PROLEARS study, which reported increased mortality in macroadenomas but not microadenomas, in a cohort smaller than ours (196 microadenomas and 54 macroadenomas) [8]. The dose-response relationship between tumor size quartiles and SMR (0.554, 0.901, 1.187, and 1.573 in Q1-Q4, respectively) supports stratification by tumor size in future prolactinoma outcome research.

Several limitations merit consideration. First, restriction of the clustering analysis to patients with available tumor size is a key limitation, as tumor size was more often documented in more recent calendar years and among surgically treated patients. Although between-cluster heterogeneity was robust to imputation, the clustering solution itself was derived from patients with preferentially documented tumor size, potentially underrepresenting medically managed prolactinomas. Second, SEER is restricted to the United States, limiting generalizability. Third, diagnostic misclassification and reporting bias cannot be excluded. Fourth, SEER lacks data on dopamine agonist treatment, prolactin levels, tumor invasion, hypopituitarism, and comorbidities, making residual confounding inevitable. Fifth, the modest number of deaths warrants caution, particularly in SAYF. Sixth, because the analysis compares observed with matched expected mortality rather than modelling competing risks, the estimates should be read as relative comparisons with the general population rather than disease effect. Finally, exclusion of patients aged ≥ 90 years, necessitated by SEER data structure, may have produced a conservative estimate of excess mortality, especially in LOOM, which was disproportionately composed of older patients.

In conclusion, prolactinoma evaluated in a population-based cancer registry may be associated with statistically significant excess all-cause mortality. A data-driven clustering approach identified two population-level profiles with divergent survival patterns: an older, predominantly male profile with larger tumors consistently demonstrated excess mortality, whereas a younger, predominantly female profile with smaller tumors showed directionally lower mortality relative to the general population, although the latter effect was attenuated and no longer statistically significant after accounting for missing tumor size. These findings describe observed mortality patterns rather than causal relationships and should be interpreted as hypothesis-generating rather than definitive. This work provides a population-level description of survival heterogeneity and highlights the need for further investigation into the biological and contextual factors underlying these differences.

## Supplementary Information

Below is the link to the electronic supplementary material.

Supplementary Material 1 (DOCX 1.31 MB)

## Data Availability

All data used are publicly available (https://seer.cancer.gov/).
