# Supplementary material for: Importance of clinical profiling to determine excess mortality in prolactinomas: insights from a large, registry-based cohort
Source: Pituitary. 2026 Sep 3;29(5):153. doi: 10.1007/s11102-026-01739-w (PMC13541857; doi:10.1007/s11102-026-01739-w)
Supplement: Supplementary file 1 [file 11102_2026_1739_MOESM1_ESM.docx]

**SUPPLEMENTARY MATERIAL**

**Importance of clinical profiling to determine excess mortality in prolactinomas: insights from a large, registry-based cohort**

*Authors*

Tarik Ziyad Tarik Shwaish (MD) ^1,2^, Davide Gori (MD, PhD) ^2^, Claudio Sartori (PhD) ^3^, Federica Guaraldi (MD, PhD) ^4^

*Affiliations*

^1^ Department of Medical Sciences, Division of Endocrinology, Diabetes and Metabolism, University of Turin, 10126 Turin, Italy.

^2^ Department of Biomedical and Neuromotor Sciences, University of Bologna, 40126 Bologna, Italy.

^3^ Department of Computer Science and Engineering, University of Bologna, 40126 Bologna, Italy.

^4^ Pituitary Unit, IRCCS Istituto delle Scienze Neurologiche di Bologna, 40139 Bologna, Italy.

*Correspondence*

Prof. Davide Gori

Department of Biomedical and Neuromotor Sciences

Alma Mater Studiorum - University of Bologna

Via San Giacomo, 12, 40126 Bologna

Phone: +39 051 209 4802; email: [davide.gori4@unibo.it](mailto:davide.gori4@unibo.it)

*Journal*

Pituitary (Springer)

**Table S1.** Comparison of patients with tumor size missing (n = 897) and tumor size known (n = 2,481).

Continuous variables are described as median (interquartile range) and compared using Mann-Whitney U test. Categorical variables are described as percentage (frequency) and compared using Fisher’s exact test.

| **Variable** | **Group** | **Tumor size missing (26.6%**  **[n = 897])** | **Tumor size known (73.4%**  **[n = 2,481])** | **SMD** | ***p*-value** |
| --- | --- | --- | --- | --- | --- |
| Age (years) |  | 37.0  (29.0-48.0) | 35.0  (28.0-48.0) | -0.073 | 0.027 |
| Surgery (received) ^a^ |  | 10.4%  (92/886) | 15.6%  (386/2,472) | 0.156 | <0.001 |
| Radiation (received) |  | 0.8%  (7/897) | 0.7%  (17/2,481) | -0.011 | 0.817 |
| Year of diagnosis |  | 2013  (2009-2017) | 2015  (2011-2019) | 0.342 | <0.001 |
| Race ^b^ | White | 50.9%  (444/872) | 51.8% (1257/2,427) | 0.018 | 0.104 |
|  | Hispanic | 23.3%  (203/872) | 23.5%  (571/2,427) | 0.006 |  |
|  | Black | 17.0%  (148/872) | 13.7%  (332/2,427) | -0.092 |  |
|  | Asian or Pacific Islander | 7.6%  (66/872) | 9.4%  (229/2,427) | 0.067 |  |
|  | American Indian/Alaska Native | 1.3%  (11/872) | 1.6%  (38/2,427) | 0.026 |  |
| GTR (yes, only for operated patients) ^c^ |  | 13.6%  (12/88) | 11.0%  (42/383) | 0.081 | 0.462 |
| Sex (male) |  | 34.4%  (309/897) | 38.2%  (947/2,481) | 0.077 | 0.048 |

^a^ Excluding 20 records (0.6%) with unknown surgical history

^b^ Excluding 79 patients (2.3%) with unknown (non-Hispanic) race

^c^ Excluding 7 patients who received surgery, but resection margins were unknown

**Table S2.** Causes of death among the study population, stratified by tumor size missingness status. Deaths are presented as percentage (frequency). *The table is continued on the next page.*

|  | **Complete cohort**  **(n = 3,378 patients,**  **168 deaths)** | **Tumor size missing**  **(n = 897 patients,**  **56 deaths)** | **Tumor size known**  **(n = 2,481 patients, 112 deaths)** |
| --- | --- | --- | --- |
| **Cardiovascular-pulmonary** |  |  |  |
| Ischemic heart disease | 8.9% (15/168) | 7.1% (4/56) | 9.8% (11/112) |
| Other and unspecified disorders of the circulatory system | 6.0% (10/168) | 10.7% (6/56) | 3.6% (4/112) |
| Cerebrovascular diseases | 4.8% (8/168) | 1.8% (1/56) | 6.2% (7/112) |
| Hypertensive disease | 2.4% (4/168) | 1.8% (1/56) | 2.7% (3/112) |
| Diseases of arteries, arterioles and capillaries | 0.6% (1/168) | 0.0% (0/56) | 0.9% (1/112) |
| Pulmonary heart disease and diseases of pulmonary circulation | 0.6% (1/168) | 1.8% (1/56) | 0.0% (0/112) |
| Chronic obstructive pulmonary disease and allied conditions | 4.2% (7/168) | 3.6% (2/56) | 4.5% (5/112) |
| **Neoplastic** |  |  |  |
| Brain, CNS other and intracranial gland (benign and borderline) | 6.5% (11/168) | 5.4% (3/56) | 7.1% (8/112) |
| Miscellaneous neoplasms | 3.0% (5/168) | 1.8% (1/56) | 3.6% (4/112) |
| Breast | 3.0% (5/168) | 5.4% (3/56) | 1.8% (2/112) |
| Lung and bronchus | 2.4% (4/168) | 0.0% (0/56) | 3.6% (4/112) |
| Pancreas | 2.4% (4/168) | 1.8% (1/56) | 2.7% (3/112) |
| Prostate | 1.8% (3/168) | 1.8% (1/56) | 1.8% (2/112) |
| Urinary bladder | 1.2% (2/168) | 0.0% (0/56) | 1.8% (2/112) |
| Myeloproliferative neoplasms and myelodysplastic syndromes | 1.2% (2/168) | 0.0% (0/56) | 1.8% (2/112) |
| Brain (malignant) | 1.2% (2/168) | 0.0% (0/56) | 1.8% (2/112) |
| Acute myeloid leukemias | 1.2% (2/168) | 3.6% (2/56) | 0.0% (0/112) |
| Intrahepatic bile duct | 1.2% (2/168) | 0.0% (0/56) | 1.8% (2/112) |
| Benign and borderline: all other sites | 1.2% (2/168) | 1.8% (1/56) | 0.9% (1/112) |
| Non-Hodgkin lymphomas | 0.6% (1/168) | 0.0% (0/56) | 0.9% (1/112) |
| Melanoma of the skin | 0.6% (1/168) | 0.0% (0/56) | 0.9% (1/112) |
| Stomach | 0.6% (1/168) | 0.0% (0/56) | 0.9% (1/112) |
| Hodgkin lymphomas | 0.6% (1/168) | 0.0% (0/56) | 0.9% (1/112) |
| Urethra | 0.6% (1/168) | 0.0% (0/56) | 0.9% (1/112) |
| Other leukemias | 0.6% (1/168) | 0.0% (0/56) | 0.9% (1/112) |
| Plasma cell neoplasms and immunoproliferative diseases | 0.6% (1/168) | 0.0% (0/56) | 0.9% (1/112) |
| Bones and joints | 0.6% (1/168) | 1.8% (1/56) | 0.0% (0/112) |
| Anus, anal canal and anorectum | 0.6% (1/168) | 1.8% (1/56) | 0.0% (0/112) |
| Colon and rectum (excluding appendix) | 0.6% (1/168) | 1.8% (1/56) | 0.0% (0/112) |
| Liver | 0.6% (1/168) | 1.8% (1/56) | 0.0% (0/112) |
| Corpus | 0.6% (1/168) | 1.8% (1/56) | 0.0% (0/112) |
| **Miscellaneous conditions** |  |  |  |
| Diabetes mellitus | 3.6% (6/168) | 3.6% (2/56) | 3.6% (4/112) |
| Alzheimer’s | 3.0% (5/168) | 1.8% (1/56) | 3.6% (4/112) |
| Nephritis, nephrotic syndrome, and nephrosis | 1.8% (3/168) | 1.8% (1/56) | 1.8% (2/112) |
| Symptoms, signs and ill-defined conditions | 1.8% (3/168) | 1.8% (1/56) | 1.8% (2/112) |
| Chronic liver disease and cirrhosis | 1.2% (2/168) | 0.0% (0/56) | 1.8% (2/112) |
| Congenital anomalies | 0.6% (1/168) | 0.0% (0/56) | 0.9% (1/112) |
| **External cause** |  |  |  |
| Accidents and adverse effects | 3.6% (6/168) | 1.8% (1/56) | 4.5% (5/112) |
| Suicide and self-inflicted injury | 1.2% (2/168) | 1.8% (1/56) | 0.9% (1/112) |
| Homicide and legal intervention | 1.2% (2/168) | 1.8% (1/56) | 0.9% (1/112) |
| **Infectious** |  |  |  |
| COVID (2020+ only) | 3.0% (5/168) | 1.8% (1/56) | 3.6% (4/112) |
| Septicemia | 1.2% (2/168) | 1.8% (1/56) | 0.9% (1/112) |
| Pneumonia and influenza | 0.6% (1/168) | 1.8% (1/56) | 0.0% (0/112) |
| Other infectious and parasitic diseases including HIV | 0.6% (1/168) | 0.0% (0/56) | 0.9% (1/112) |
| **Other and unknown** |  |  |  |
| Other cause of death | 12.5% (21/168) | 19.6% (11/56) | 8.9% (10/112) |
| Death certificate not available or death certificate available but no cause of death | 3.6% (6/168) | 3.6% (2/56) | 3.6% (4/112) |

**Table S3.** Stratified standardized mortality ratio analysis in the full cohort (n = 3,378)

| **Variable** | **Group** | **n** | **Deaths (n)** | | **SMR** | | **Heterogeneity *p*-value** |
| --- | --- | --- | --- | --- | --- | --- | --- |
|  |  |  | **Observed** | **Expected** | **Estimate (95%CI)** | ***p*-value** |  |
| Overall | All prolactinomas | 3,378 | 168 | 138.833 | 1.210 (1.034-1.408) | 0.018 |  |
| Sex | Male | 1,256 | 101 | 89.909 | 1.123 (0.915-1.365) | 0.266 | 0.209 |
|  | Female | 2,122 | 67 | 48.924 | 1.369 (1.061-1.739) | 0.016 |  |
| Tumor size ^a^ | Q1 (1-6 mm) | 802 | 9 | 16.252 | 0.554 (0.253-1.051) | 0.076 | 0.015 |
|  | Q2 (6-10 mm) | 497 | 13 | 14.433 | 0.901 (0.480-1.540) | 0.839 |  |
|  | Q3 (10-22 mm) | 590 | 32 | 26.952 | 1.187 (0.812-1.676) | 0.377 |  |
|  | Q4 (22-115 mm) | 592 | 58 | 36.862 | 1.573 (1.195-2.034) | 0.002 |  |
| Radiation | Received | 24 | 4 | 1.222 | 3.273 (0.892-8.379) | 0.072 | 0.046 |
|  | Not received | 3,354 | 164 | 137.610 | 1.192 (1.016-1.389) | 0.031 |  |
| Surgery ^b^ | No surgery | 2,880 | 130 | 115.071 | 1.130 (0.944-1.341) | 0.182 | 0.067 |
|  | STR | 417 | 28 | 20.035 | 1.398 (0.929-2.020) | 0.107 |  |
|  | GTR | 54 | 7 | 2.663 | 2.628 (1.057-5.415) | 0.039 |  |
| Age | Q1 (18-28 years) | 893 | 5 | 7.225 | 0.692 (0.225-1.615) | 0.546 | 0.094 |
|  | Q2 (28-36 years) | 855 | 12 | 9.705 | 1.236 (0.639-2.160) | 0.541 |  |
|  | Q3 (36-48 years) | 829 | 18 | 23.003 | 0.782 (0.464-1.237) | 0.349 |  |
|  | Q4 (48-89 years) | 801 | 133 | 98.899 | 1.345 (1.126-1.594) | 0.001 |  |
| Race/ethnicity ^c^ | White | 1,701 | 92 | 87.230 | 1.055 (0.850-1.293) | 0.638 | 0.002 |
|  | Black | 480 | 27 | 22.419 | 1.204 (0.794-1.752) | 0.383 |  |
|  | American Indian/Alaska Native | 49 | 2 | 3.279 | 0.610 (0.074-2.203) | 0.727 |  |
|  | Asian or Pacific Islander | 295 | 15 | 5.267 | 2.848 (1.594-4.697) | <0.001 |  |
|  | Hispanic | 774 | 32 | 18.326 | 1.746 (1.194-2.465) | 0.005 |  |
| Menopause (female only) | Premenopause (<52 years) | 1,926 | 29 | 23.829 | 1.217 (0.815-1.748) | 0.337 | 0.375 |
|  | Postmenopause (≥52 years) | 196 | 38 | 25.094 | 1.514 (1.072-2.078) | 0.020 |  |

^a^ Excluding 897 patients (26.6%) with missing tumor size

^b^ Excluding 27 patients (0.8%) with missing surgical data: 20 (0.6%) with unknown surgical history and 7 (0.2%) known to have received surgery but with unknown resection margins

^c^ Excluding 79 patients (2.3%) with unknown (non-Hispanic) race

**Table S4.** Sensitivity analyses results

| **Analysis** | **n** | **ARI** | **LOOM SMR** | | **SAYF SMR** | | **Adjusted EMR** | |
| --- | --- | --- | --- | --- | --- | --- | --- | --- |
|  |  |  | **Estimate**  **(95% CI)** | ***p*-value** | **Estimate**  **(95% CI)** | ***p*-value** | **Estimate**  **(95% CI)** | ***p*-value** |
| Clustering re-run within the clustering cohort |  |  |  |  |  |  |  |  |
| Exclusion of 8 prolactinoma-attributable deaths | 2,473 | >0.99 | 1.261  (1.020-1.541) | 0.032 | 0.476  (0.218-0.904) | 0.019 | 2.679  (1.325–5.420) | 0.006 |
| Tumor size Winsorized at the 99th percentile | 2,481 | 0.85 | 1.390  (1.134-1.688) | 0.002 | 0.473  (0.227-0.870) | 0.012 | 2.969  (1.527-5.770) | 0.001 |
| Clustering based on age + tumor size only, with fuzzifier value *m*=3.7 | 2,481 | 0.83 | 1.361  (1.110-1.653) | 0.003 | 0.511  (0.245-0.940) | 0.027 | 2.679  (1.377-5.212) | 0.004 |
| Clustering based on age + tumor size only, with fuzzifier value *m*=6.8 | 2,481 | 0.84 | 1.374  (1.123-1.665) | 0.002 | 0.425  (0.184-0.838) | 0.009 | 3.265  (1.571-6.786) | 0.002 |
| Original centroids applied to 30 imputed full cohort datasets (pooled estimates) | 3,781 | - | 1.316  (1.120-1.547) | <0.001 | 0.769  (0.497-1.188) | 0.236 | 1.763  (1.091-2.849) | 0.021 |

**Fig. S1** Scatter plot of the study cohort in the age at diagnosis (years) × tumor size (mm) space. Each symbol represents one patient: circles indicate females and triangles indicate males. The background color gradient represents the difference in fuzzy membership scores (µ) between clusters (µ_LOOM_ − µ_SAYF_), estimated via cubic triangular interpolation; warm colors (orange) indicate dominant SAYF membership, cool colors (blue) indicate dominant LOOM membership. The axes are scaled based on percentile transformation to handle skewedness; ticks on the x-axis (tumor size) and y-axis (age at diagnosis) correspond to 40-mm and 10-year intervals, respectively. LOOM = Large or Old, Male-predominant; SAYF = Small and Young, Female-predominant.
